# Efficacy of Rg1-Oil Adjuvant on Inducing Immune Responses against *Bordetella bronchiseptica* in Rabbits

**DOI:** 10.1155/2021/8835919

**Published:** 2021-01-28

**Authors:** Xiao Chenwen, Ji Quanan, Huang Yee, Liu Yan, Wang Jiaoyu, Wei Qiang, Qiao Litao, Nan Li, Bao Guolian

**Affiliations:** ^1^Institute of Animal Husbandry and Veterinary Science, Zhejiang Academy of Agricultural Sciences, Hangzhou, Zhejiang 310021, China; ^2^Institute of Plant Protection and Microbiology, Zhejiang Academy of Agricultural Science, Hangzhou 310021, China; ^3^Department of Ultrasound, PKUCare Luzhong Hospital, Zibo, Shandong 255400, China

## Abstract

*Bordetella bronchiseptica* (*B*. *bronchiseptica*) is an obligately aerobic, oxidase- and catalase-positive, nonfermentative Gram-negative coccobacillus. This study is aimed at examining the immune effects of Rg1, Rg1 plus oil, and other common adjuvants on inactivated *B*. *bronchiseptica* vaccine in rabbits. The mechanism underlying the adjuvant effect of Rg1 plus oil on the vaccine was also explored. Rg1 (100 *μ*g) plus oil significantly improved the immune effect of *B*. *bronchiseptica* vaccine at both the humoral and cellular levels. Rg1-oil adjuvant increased the levels of IL-2 and IL-4 in rabbits after immunization. Rg1 (100 *μ*g) plus oil also significantly increased TLR2 expression and downregulated NF-*κ*B in splenocytes. Rg1-oil adjuvant may increase the levels of IL-2 and IL-4 via upregulating TLR2, thereby enhancing the immune effect of *B*. *bronchiseptica* vaccine. In conclusion, Rg1 plus oil could be used as a potential vaccine adjuvant for rabbit *B*. *bronchiseptica* vaccine.

## 1. Introduction

*Bordetella bronchiseptica* (*B*. *bronchiseptica*) is an obligately aerobic, oxidase- and catalase-positive, nonfermentative Gram-negative coccobacillus that was first identified in dogs with distemper disease in 1911 [1]. It causes infections in the respiratory tract of rabbits and acts as a precursor for secondary infection with *Pasteurella multocida* [2]. *B*. *bronchiseptica* infection in rabbits occurs all year round, but more commonly in autumn and spring. The nasal secretion from infected animals may contaminate air, food, and water, which facilitates the spread of the disease. *B*. *bronchiseptic*a infection not only induces pustular pneumonia, bronchitis, and rhinitis in adult rabbits but also leads to acute death in young animals both before and after weaning. The rapid spread and difficulties in complete eradication of this disease often result in serious economic loss [3].

Vaccination is one of the most effective interventions to prevent *B*. *bronchiseptica* infection in rabbits. However, the development of adjuvants for *B*. *bronchiseptica* vaccine is still challenging. Currently available adjuvants, such as synthetic peptide vaccine and DNA recombinant vaccine, do not meet the demands of vaccines with low immunogenicity. Extensive research on the development of novel adjuvants has been carried out. However, these adjuvants have not been widely used due to poor safety performance. Significant advances have been made to enhance the potency of currently available vaccines by incorporating adjuvants into vaccine formulations. Besides the immune-stimulating effect, adjuvants also reduce the amount of antigens needed for vaccination, thus reducing the cost of production. However, the incorporation of adjuvants into the vaccine should be carefully evaluated to ensure that no adverse side effects occur and the immunogenic effect is stimulated [4]. Ginseng, the root of Panax ginseng C.A. Meyer (Araliaceae), is a herbal tonic used in traditional Chinese medicine for over 2000 years. The adjuvant effects of ginseng saponins (ginsenosides) on immune responses in rats, mice, pigs, and cattle have been widely reported [5]. Rg1 is a ginsenoside that exhibits adjuvant activity in immune responses [6, 7]. In this work, we evaluated the immune effects of Rg1 and other common adjuvants on inactivated rabbit *B*. *bronchiseptica* vaccine and explored the adjuvant mechanism of Rg1. Our findings provided scientific support for the development of *B*. *bronchiseptica* vaccines.

## 2. Materials and Methods

### 2.1. Antigens and Adjuvants

White oil (mineral oil), alhydrogel adjuvant, and *B*. *bronchiseptica* antigen were obtained from Connaught Times Wei Biotechnology Company (Hangzhou, China). *B*. *bronchiseptica* (FX strain) was cultured through GMP-certified fermentation followed by sterilization with 0.2% formalin for 36 h. After a sterilization test, *B*. *bronchiseptica* was stored at 4°C for further analyses. Whole proteins were isolated from *B*. *bronchiseptica* for ELISA. Rg1 was purchased from Yuanye Biotechnology (Cat No. 22427-39-0, Shanghai, China). QA was kindly provided by Zhejiang University. Quil A was obtained from Desert King Chile Ltd. (Santiago, Chile). QA and Rg1 solution was analyzed for endotoxin using a Limulus Amebocyte Lysate gel-clot assay (Cat No. 118792, Zhanjiang A&C Biological Ltd., Zhanjiang, China) to ensure that the endotoxin level was <0.5 endotoxin units/mL.

### 2.2. Vaccine Preparation

Oil-based adjuvants were emulsified for 90 min using an automatic fast-sample grinding instrument (JingXin Technology, Shanghai, China) with an emulsification index of 60 hertz. This procedure was repeated twice. The components of each vaccine are shown in Tables 1 and 2.

### 2.3. Animals and Study Design

This study was approved by the Ethics Committee of the Zhejiang Academy of Agricultural Sciences (ethics protocol no. 002762) and performed in accordance with the principles and guidelines of the Zhejiang Farm Animal Welfare Council of China. Five-week-old male New Zealand rabbits (weighing 1-1.5 kg) were obtained from the Zhejiang Animal Center and allowed to acclimate for one week before experiments.

#### 2.3.1. Experiment A

Rabbits were randomly assigned into eight groups (*n* = 15 per group) and then immunized by subcutaneous injection of 1 mL of *B*. *bronchiseptica* (1.5 × 10^10^ CFU) alone, *B*. *bronchiseptica* (1.5 × 10^10^ CFU) dissolved in PBS containing 400 *μ*g Rg1, 400 *μ*g Rg1 plus 750 *μ*L oil, 750 *μ*L oil alone, 50 *μ*g QA, or 200 *μ*g Alum into the neck on Day 1 (Table 1). Blood samples were collected from the ear vein of each animal before and at 5, 10, 15, 21, and 35 days after injection and kept at 4°C. The body weight of each animal was measured before and after immunization on Days 0, 5, 10, 15, and 35. All rabbits were monitored for adverse drug reactions after immunization.

#### 2.3.2. Experiment B

Rabbits were randomly divided into six groups (*n* = 8 per group) and then subcutaneously injected with 1 mL of *B*. *bronchiseptica* (1.5 × 10^10^ CFU) alone, *B*. *bronchiseptica* (1.5 × 10^10^ CFU) dissolved in PBS containing 100, 200, or 400 *μ*g Rg1 plus 750 *μ*L oil, or 750 *μ*L oil alone into the neck on Day 1 (Table 2). Blood samples were collected from the ear vein of each rabbit before immunization and at 15 and 30 days postinjection and stored at 4°C. All rabbits were monitored for adverse drug reactions after immunization.

### 2.4. Measurement of the *B*. *bronchiseptica*-Specific IgG Level

The serum level of *B*. *bronchiseptica*-specific IgG was determined by indirect ELISA. Microtiter plate wells were purchased from Gongdong Medical Plastic Factory (Zhejiang, China). Each well was coated with 100 *μ*L of 1 *μ*g/mL *B*. *bronchiseptica* protein dissolved in 0.05 M carbonate buffer overnight at 4°C. After washing with 0.05% PBST (Tween-20), wells were blocked with 5% nonfat milk for 2 h at 37°C followed by three washes. Then, 100 *μ*L diluted serum sample (1 : 400) was added to each well in triplicate. After 1 h incubation at 37°C, a horseradish peroxidase-conjugated goat anti-rabbit antibody (1 : 5000, KLP, USA) was added to each well and incubated at 37°C for 1 h. After washing with 0.05% PBST (Tween-20), samples were incubated with 100 *μ*L of substrate solution at 37°C for 10 min. The reaction was terminated by the addition of 2 N H_2_SO_4_ (50 *μ*L per well). The optical density (OD) was detected at 450 nm using an ELISA reader.

### 2.5. Analysis of the *B*. *bronchiseptica*-Specific Antibody Titer

Serum samples collected from Experiment A were serially diluted in PBS by twofold into a 12-well plate starting with a 1 : 10 dilution. Each well contained 50 *μ*L serum in a V-shaped 96-well plate. Subsequently, samples were gently mixed with 50 *μ*L of *B*. *bronchiseptica* (a mixture of 9 mL *B*. *bronchiseptica* bacterial fluid (OD620 value = 2.5) and 100 *μ*L methylene blue) for 1 h at 37°C and then transferred to 4°C. The next day, after 10 min incubation at room temperature, the test results were recorded, including blank (PBS), negative, and positive controls.

### 2.6. Analysis of Cytokine Levels

The levels of IL-2 and IL-4 in rabbits from Experiment B were determined by ELISA (Cat Nos. ml0029781-2 and ml 027170, MLBIO, Shanghai Enzyme-Linked Biotechnology, Shanghai, China). Each well was filled with 50 *μ*L of sample or standard and then added with 100 *μ*L of horseradish peroxidase-labeled antibody. After 1 h incubation at 37°C, the plate was incubated with substrate A solution (50 *μ*L) and substrate B solution (50 *μ*L) at 37°C for 15 min in the dark. After adding 50 *μ*L of stopping solution, the OD value was measured at 450 nm.

### 2.7. Differential Blood Cell Count

Blood samples collected from Experiment A were transferred to tubes containing EDTA (Sigma-Aldrich) as anticoagulant on Days 10, 15, 21, and 35 postimmunization. Blood samples collected from Experiment B were transferred to tubes containing EDTA as anticoagulant at 15 days postimmunization. A differential blood cell count was performed using an analyzer (Sysmex, pocH-100iV, JP). The numbers of neutrophil granulocytes, intermediate cells, and lymphocytes were counted.

### 2.8. Real-Time PCR (RT-PCR)

RNA was extracted from spleen samples collected from Experiment B at 30 days postimmunization using the PureLink RNA Mini Kit (Life Technologies, USA). The concentration of total RNA was determined by the OD value at 260 nm. Reverse transcription was performed using a commercially available kit (Promega, Madison, WI, USA). Then, RT-PCR was performed to determine the expression levels of NF-*κ*B2 and TLR2. The primers used in this experiment (Table 3) were designed according to rabbit genome sequences shown in GenBank. ACTB was used as an internal control. The 2^-*ΔΔ*CT^ method was used for relative quantification. Each sample was analyzed at least twice.

### 2.9. Western Blot

Frozen spleen tissue samples were homogenized using RIPA buffer and then centrifuged at 12000 rpm for 10 min at 4°C to obtain supernatants. Protein concentration was determined by the BCA method. Samples were added with 5x SDS loading buffer, boiled at 100°C for 5 min, separated on SDS-PAGE, and finally transfected to the PVDF membrane. After blocked with 5% milk at room temperature for 1 h, the membrane was incubated with anti-TLR2 antibody (1 : 2000, Cat No. NBP2-24909, Novus) and anti-beta-actin antibody (1 : 20000, Cat No. 8226, Abcam) overnight at 4°C. After three washes with PBST, the membrane was incubated with a goat anti-mouse secondary antibody (1 : 5000, Cat No. 00001-1, Wuhan Sanying, China) (1 : 5000) at room temperature for 1 h. The same amounts of enhanced luminol reagent and oxidizing reagent were diluted in ddH_2_O and then dropped on the sealing film. The results were analyzed using a gel imaging system.

### 2.10. Immunofluorescence Analysis of NF-*κ*B

HeLa cells were cultured in 10% serum medium in a 37°C/CO_2_ incubator until 70% confluence. Then, cells were digested by trypsin, collected, and evenly plated into a 24-well plate covered with aseptic climbing tablets. Rg1 (1 mg) was dissolved in 1 mL PBS, which was then diluted in gradient with culture medium. Cells were incubated with medium containing with or without Rg1 at 10, 20, 40, 100, 200, and 400 *μ*g/mL for 2 h. After treatment, cells were fixed with 4% paraformaldehyde for 20 min, followed by 15 min incubation with 0.5% Triton X-100 (prepared by 1x PBS) at room temperature. After washing, 50 *μ*L of diluted ProteinFind® anti-NF-*κ*B p65 mouse monoclonal antibody (1 : 100, Cat No. HA106) was added to each cell climbing tablet and incubated at 4°C overnight. Subsequently, climbing tablets were gently washed with 1x PBST for 5 min and incubated with a fluorescent ProteinFind® secondary anti-rabbit IgG (H+L) antibody conjugated to AF488 (1 : 100, Cat No. HS131) at 37°C for 1 h. After three washes with 1x PBST, samples were incubated with 40 *μ*L of Hoechst 33342 for 15 min to stain the nuclei. Then, the cell climbing sheet was sealed with sealing liquid and cells were photographed under a fluorescence microscope.

### 2.11. Statistical Analysis

Data were shown as the mean ± SD. The differences among groups were compared using Tukey's HSD test and one-way ANOVA. A *P* value of <0.05 was considered statistically significant.

## 3. Results

### 3.1. Measurements of the *B*. *bronchiseptica*-Specific Serum IgG Level and Antibody Agglutination

**Figure 1:**
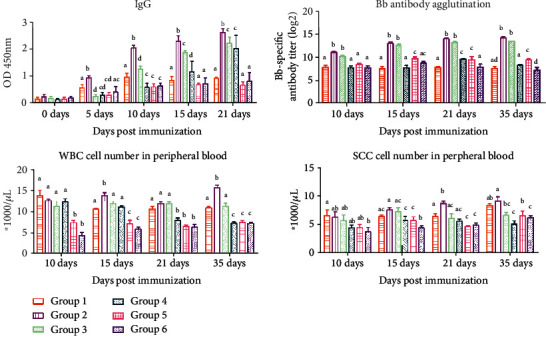
The serum level of whole bacterial protein-specific IgG antibody in rabbits from Experiment A was detected by indirect ELISA. An agglutination test was performed to measure *B*. *bronchiseptica*-specific antibody titer. Blood samples were collected from each group of rabbits as anticoagulants at 10, 15, 21, and 35 days postimmunization for the detection of WBC and SCC.

In Experiment A, the serum levels of *B*. *bronchiseptica*-specific IgG in rabbits after immunization were monitored over time (Figure 1) (S-OD450nm). The IgG concentration started to increase at 5 days postinjection in all groups. From Days 10-21, the serum IgG level in rabbits immunized with *B*. *bronchiseptica* dissolved in Rg1-oil adjuvant was significantly increased compared to that in other adjuvant groups (*P* < 0.05). On Days 10 and 15, the serum IgG concentration in the oil group was significantly higher than that in other adjuvant groups (*P* < 0.05), except the Rg1-oil group.

Compared to other adjuvant groups, the antibody agglutination in animals immunized with *B*. *bronchiseptica* was significantly enhanced by Rg1-oil adjuvant and oil alone from Days 10 to 35 (*P* < 0.05). No significant difference was observed between the Rg1-oil and oil groups (Figure 1) (S-Bb antibody agglutination).

**Figure 2:**
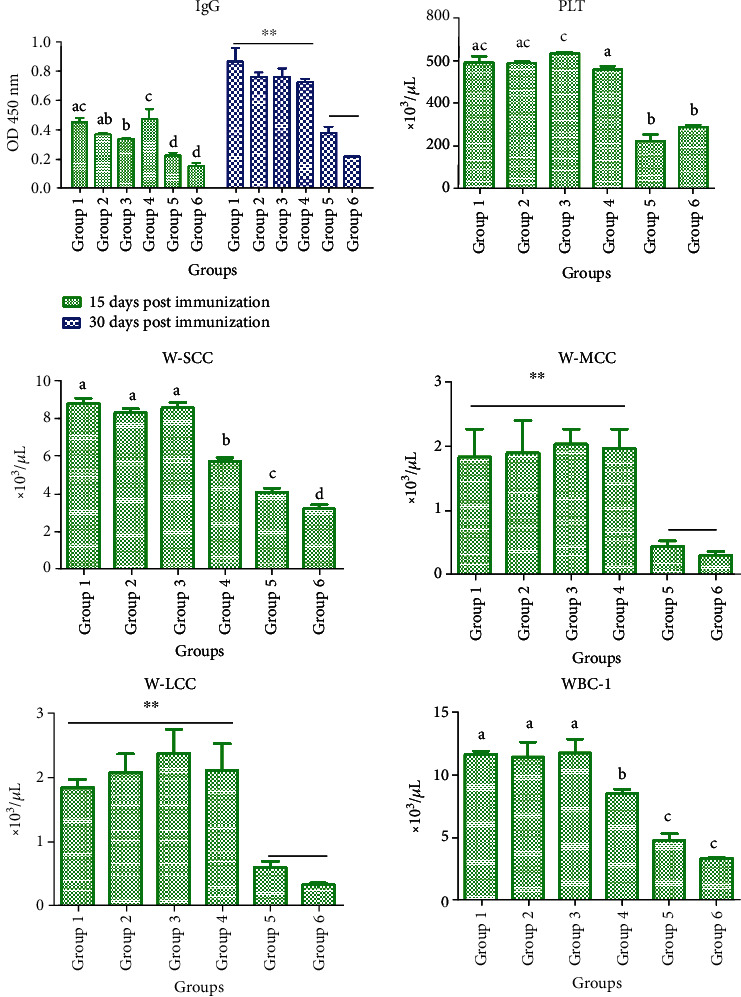
The serum level of whole bacterial protein-specific IgG antibody in rabbits from Experiment B was detected by indirect ELISA at 15 and 30 days postinjection. Blood samples were collected as anticoagulants at 15 days after injection for the detection of PLT, SCC, MCC, LCC, and WBC.

In Experiment B, the serum levels of *B*. *bronchiseptica*-specific IgG in rabbits after immunization were monitored over time (Figure 2) (S-IgG). Compared to other adjuvant groups, the serum IgG level in rabbits immunized with *B*. *bronchiseptica* was effectively increased by Rg1 (100, 200, and 400 *μ*g) plus oil and oil alone from Days 15 to 30 (*P* < 0.05).

### 3.2. Differential Blood Cell Count

In Experiment A, the number of WBC in rabbits immunized with *B*. *bronchiseptica* was significantly increased by Rg1-oil adjuvant compared to other adjuvant groups (*P* < 0.05) on Days 15 and 35 (Figure 1) (S-WBC cell detection). On Day 21, the number of SCC in rabbits immunized with *B*. *bronchiseptica* dissolved in Rg1-oil adjuvant was significantly increased compared to that in other adjuvant groups (*P* < 0.05, Figure 1) (S-SCC cell detection).

In Experiment B, the numbers of PLT, MCC, and LCC in rabbits immunized with *B*. *bronchiseptica* were significantly increased by Rg1 (100, 200, and 400 *μ*g) plus oil and oil alone compared to other adjuvant groups (*P* < 0.05) on Day 15 (Figure 2) (S-PLT, S-W-MCC, and S-W-LCC). On Day 15, the numbers of SCC and WBC were also significantly increased by Rg1 (100, 200, and 400 *μ*g) plus oil compared to other adjuvant groups (*P* < 0.05, Figure 2) (S-W-SCC, S-WBC-1).

### 3.3. Effect of Rg1 on Growth Performance

**Figure 3:**
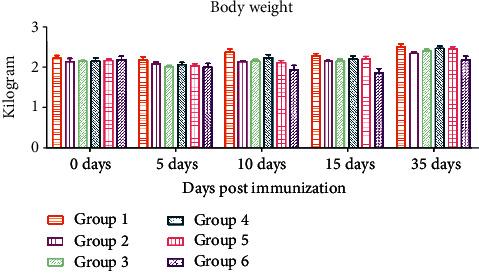
The body weight of rabbits in Experiment A was measured before and after immunization on Days 0, 5, 10, 15, and 35.

The body weight of all groups was measured before and after immunization on Days 0, 5, 10, 15, and 35. No significant difference was observed among different groups (Figure 3) (S-Body weight).

### 3.4. Measurement of Cytokine Levels

**Figure 4:**
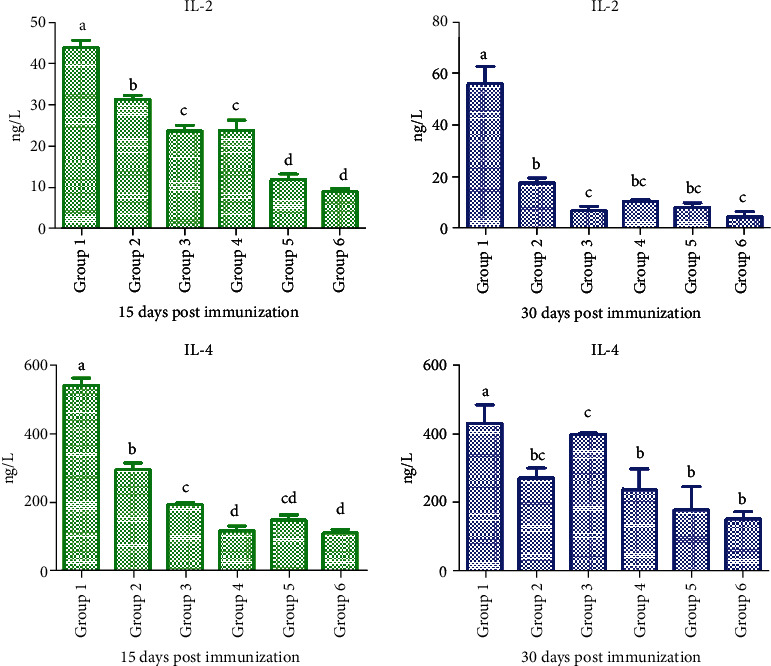
The levels of IL-2 and IL-4 in rabbits from Experiment B were assessed by ELISA at 15 and 30 days postimmunization.

The cytokine levels after immunization in Experiment B were monitored over time (Figure 4). The concentrations of IL-2 and IL-4 in the Rg1 (100 *μ*g) plus oil group were significantly higher than those in other adjuvant groups on Days 15 and 30 (*P* < 0.05, Figure 4) (S-IL-2 15 days postimmunization; S-IL-2 35 days postimmunization; S-IL-4 15 days postimmunization; and S-IL-4 35 days postimmunization).

### 3.5. RT-PCR and Western Blot

**Figure 5:**
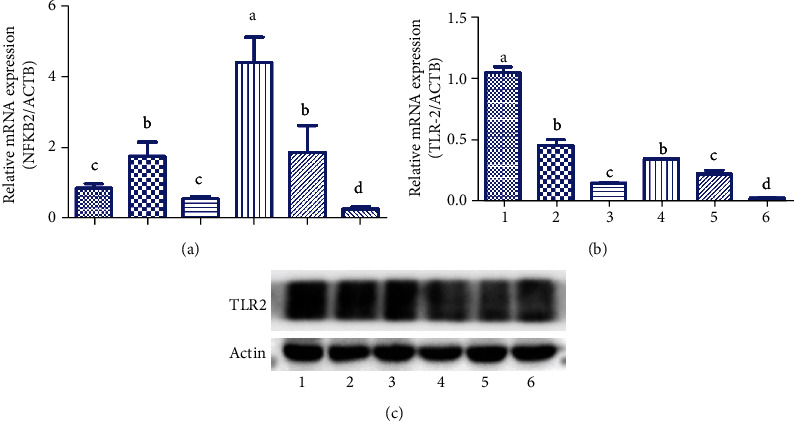
The mRNA and protein expressions of NF-*κ*B2 and TLR2 in the spleen. Total RNA was isolated from the spleen samples collected at 30 days postimmunization. The spleen tissues were homogenized using RIPA buffer.

Total RNA and protein were extracted from spleen tissue samples from rabbits in Experiment B at 30 days postimmunization. The mRNA expression of NF-*κ*B2 in the oil group was significantly higher compared to that in other groups (*P* < 0.05) on Day 30 after immunization (Figure 5) (S-NF-*κ*B RT-PCR). The protein levels of NF-*κ*B2 in the Rg1 (100 *μ*g) plus oil and antigen groups were significantly higher compared to those in others on Day 30 (*P* < 0.05), except the oil group (Figure 5(a)). The Rg1-oil (100 *μ*g) group showed significantly increased TLR2 expression on Day 30 compared with other groups (*P* < 0.05, Figure 5(b)) (S-TLR2 RT-PCR). The protein level of TLR2 in the Rg1-oil (100, 200, and 400 *μ*g) group was also higher compared to that in other groups on Day 30 (Figure 5(c)).

### 3.6. Immunofluorescence Analysis of NF-*κ*B

**Figure 6:**
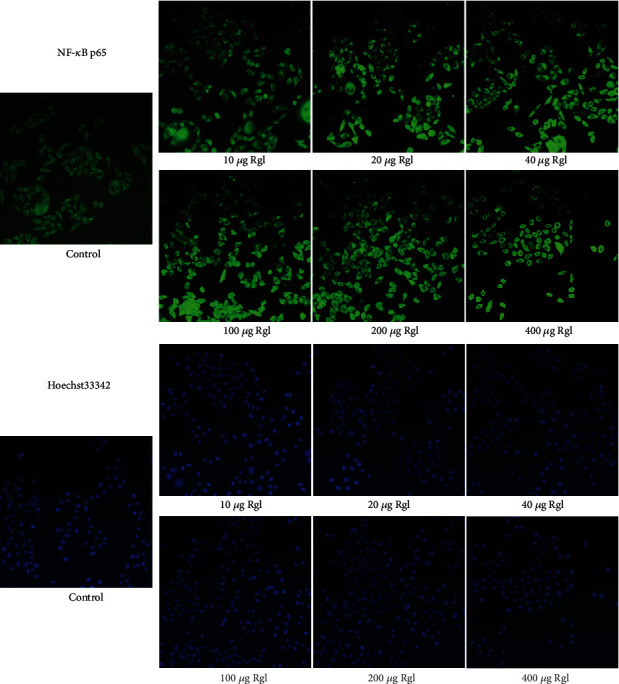
Rg1 (1 mg) was dissolved in 1 mL PBS and then diluted in gradient with culture medium. Cells were incubated with medium containing with or without Rg1 at 10, 20, 40, 100, 200, and 400 *μ*g/mL for 2 h.

The expression of NF-*κ*B in HeLa cells was dose-dependently increased in the presence of Rg1 (10-200 *μ*g), while decreased when exposed to Rg1 at 400 *μ*g (Figure 6). The expression of Hoechst 33342 showed the same change when compared with the immunofluorescence staining of NF-*κ*B (Figure 6).

## 4. Discussion

The rapid spread of *B*. *bronchiseptica* infection often leads to huge economic loss [8]. The application of attenuated *B*. *bronchiseptica* vaccines is limited due to safety issues during storage and transportation. Inactivated vaccines are safe, but the immunogenicity of *B*. *bronchiseptica* antigens is low [8]. Therefore, it is urgent to find an effective vaccine adjuvant for *B*. *bronchiseptica* vaccine. Since the original report by Espinet et al. [9] showing that saponins can be used as a vaccine adjuvant, considerable efforts have been expended to evaluate the adjuvant activity of saponins. On the one hand, saponin-based adjuvant stimulates cell-mediated immune responses and enhances antibody production at low doses. On the other hand, undesirable side effects caused by some saponins limited their application as vaccine adjuvants [10, 11]. Panax ginseng is a tonic medicine commonly used in Asian countries. Rg1 extracted from ginseng shows low hemolytic activity [12] and high immunomodulating activity [6, 13] and therefore could be used as an adjuvant for veterinary vaccines.

The syringe extrusion method has a good background for immunization [14]. The water-in-oil emulsion is prepared using syringe extrusion, vortex, or high-speed homogenization for laboratory and clinical peptide-based vaccination. In this study, we showed a synergistic effect between Rg1 and oil, consistent with previous studies showing that a mixture of saponins and oil improved the immune effect of vaccines [15, 16]. We also evaluated the effects of other adjuvants on inactivated *B*. *bronchiseptica* vaccine. The results indicated that ELISA was more sensitive than the agglutination test. The serum antibody level began to increase in all groups of rabbits since Day 5 postimmunization. The Rg1 (400 *μ*g) plus oil group showed a significantly increased antibody level and antibody titer compared to other groups, indicating the synergistic effect of Rg1-oil adjuvant on boosting humoral responses in rabbits. Moreover, the cellular immune test showed that Rg1, Rg1-oil, oil, and QA groups had a higher number of WBC compared to the aluminum glue and control groups since Day 10 postimmunization, but no significant difference was observed among Rg1, Rg1-oil, oil, and QA groups. At 15 and 35 days postimmunization, the number of WBC in the Rg1-oil group was significantly higher than that in other groups, suggesting that Rg1-oil adjuvant significantly improved cellular immune responses in rabbits. At 21 days postimmunization, the Rg1-oil group showed a significantly higher number of SCC compared to other groups. On Day 35 after immunization, the Rg1-oil group showed significantly more SCC than other groups (except the Rg1 group). Previous evidence demonstrated that Rg1 increased the proportion of T helper cells among all T cells and promoted the expression of IL-4 and IL-2 in murine splenocytes [17]. In Experiment B, the levels of IL-4 and IL-2 in the Rg1 (100 *μ*g) plus oil group were significantly higher than those in other groups, while no significant difference was observed between the oil group and other groups, indicating that Rg1 in Rg1-oil adjuvant promoted the production of IL-4 and IL-2 in immunized rabbits.

Rg1 usually exerts an inhibitory effect on NF-*κ*B in inflammation. For example, Rg1 extracted from white ginseng plays an anti-inflammatory role by reducing the expression of NF-*κ*B [18]. It was also found that Rg1 effectively suppressed allergic airway inflammation of asthma partly through the TNF-*α*/NF-*κ*B pathway [19]. Our results showed that the expression of NF-*κ*B in the Rg1 (100-400 *μ*g) plus oil group was significantly lower than that in the oil group, implying that Rg1-oil adjuvant downregulated NF-*κ*B in noninflammatory condition. The *in vitro* study demonstrated that Rg1 promoted the expression of NF-*κ*B in HeLa cells at doses between 10 *μ*g and 200 *μ*g but inhibited NF-*κ*B expression at 400 *μ*g, suggesting that Rg1 had an inhibitory effect on HeLa cells. These findings were also consistent with the RT-PCR results, which showed the splenic expression of NF-*κ*B in the Rg1 (100-400 *μ*g) plus oil group. TLRs are key transmembrane pattern recognition receptors that play an important role in initiating immune responses against invading microbial pathogens. Our data showed that Rg1 (100 *μ*g) plus oil significantly increased the expression of TLR2 in splenocytes, which was consistent with a previous report [20]. It has also been shown that active components of Panax ginseng possess a putative TLR ligand in different cell models [21, 22].

## 5. Conclusion

Rg1 (100 *μ*g) plus oil significantly improved the immune effect of rabbit *B*. *bronchiseptica* vaccine at both the humoral and cellular levels. Rg1-oil adjuvant increased the production of IL-2 and IL-4 in rabbits after immunization. Rg1 (100 *μ*g) plus oil significantly upregulated TLR2 expression but decreased the level of NF-*κ*B in splenocytes. Rg1-oil adjuvant may increase the levels of IL-2 and IL-4 by upregulating TLR2, thus enhancing the immune effect of *B*. *bronchiseptica* vaccine. Thus, Rg1-oil adjuvant could be used as a potential vaccine adjuvant for rabbit *B*. *bronchiseptica* vaccine.

## Figures and Tables

**Table 1:** Adjuvant vaccines used in Experiment A.

No.	Groups	Preparation each dose	
1	1	Rg1 (400 *μ*g) in PBS 500 *μ*L	1.5 × 10^10^ CFU in PBS 500 *μ*L
2	2	750 *μ*L oil+10 *μ*L Tween+Rg1 (400 *μ*g)	1.5 × 10^10^ CFU in PBS 500 *μ*L
3	3	750 *μ*L oil+10 *μ*L Tween	1.5 × 10^10^ CFU in PBS 500 *μ*L
4	4	50 *μ*g QA in PBS 500 *μ*L	1.5 × 10^10^ CFU in PBS 500 *μ*L
5	5	Alum (200 *μ*g) in PBS 500 *μ*L	1.5 × 10^10^ CFU in PBS 500 *μ*L
6	6	No adjuvant	1.5 × 10^10^ CFU in PBS 500 *μ*L

**Table 2:** Adjuvant vaccines used in Experiment B.

No.	Groups	Preparation each dose	
1	Rg1+oil	750 *μ*L oil+10 *μ*L Tween+Rg1 (100 *μ*g)	1.5 × 10^10^ CFU in PBS 250 *μ*L
2	Rg1+oil	750 *μ*L oil+10 *μ*L Tween+Rg1 (200 *μ*g)	1.5 × 10^10^ CFU in PBS 250 *μ*L
3	Rg1+oil	750 *μ*L oil+10 *μ*L Tween+Rg1 (400 *μ*g)	1.5 × 10^10^ CFU in PBS 250 *μ*L
4	Oil	750 *μ*L oil+10 *μ*L Tween	1.5 × 10^10^ CFU in PBS 250 *μ*L
5	No adjuvant		1.5 × 10^10^ CFU in PBS 250 *μ*L
6	PBS	1000 *μ*L	1.5 × 10^10^ CFU in PBS 250 *μ*L

**Table 3:** Sequences of primers used in RT-PCR.

Gene	Sequence
NF-*κ*B2-F	CTGGGTGTCCTACACGTGAC
NF-*κ*B2-R	GATGGGCTGGGAGATAACGG
TLR2-F	CGTGTCAGGTCAGTCAGCTT
TLR2-R	ACCCTCTGGTACTCCGTCTC
ACTB-F	GTGCTTCTAGGCGGACTGTT
ACTB-R	TCGGCCACATTGCAGAACTT

## Data Availability

Data could be found in supplemental files.

## Supplementary Materials

Supplementary Materials Concise supplementary material description: W-SCC: in Experiment B (Figure 2). W-MCC: in Experiment B (Figure 2). W-LCC: in Experiment B (Figure 2). WBC-1: in Experiment B (Figure 2). SCC cell detection: in Experiment A (Figure 1). PLT: in Experiment B (Figure 2). OD450nm: in Experiment A (Figure 1). IL-4 35 days postimmunization: in Experiment B (Figure 4). IL-2 35 days postimmunization: in Experiment B (Figure 4). Body weight: in Experiment A (Figure 3). IL-4 15 days postimmunization: in Experiment B (Figure 4). IL-2 15 days postimmunization: in Experiment B (Figure 4). IgG: in Experiment B (Figure 2). WBC cell detection: in Experiment A (Figure 1). Bb antibody agglutination: in Experiment A (Figure 1).
