# Supplementary material for: Efficacy of Rg1-Oil Adjuvant on Inducing Immune Responses against Bordetella bronchiseptica in Rabbits
Source: J Immunol Res. 2021 Jan 28;2021:8835919. doi: 10.1155/2021/8835919 (PMC7864750; doi:10.1155/2021/8835919)
Supplement: Supplementary Materials — Concise supplementary material description: W-SCC: in Experiment B (Figure 2). W-MCC: in Experiment B (Figure 2). W-LCC: in Experiment B (Figure 2). WBC-1: in Experiment B (Figure 2). SCC cell detection: in Experiment A (Figure 1). PLT: in Experiment B (Figure 2). OD450nm: in Experiment A (Figure 1). IL-4 35 days postimmunization: in Experiment B (Figure 4). IL-2 35 days postimmunization: in Experiment B (Figure 4). Body weight: in Experiment A (Figure 3). IL-4 15 days postimmunization: in Experiment B (Figure 4). IL-2 15 days postimmunization: in Experiment B (Figure 4). IgG: in Experiment B (Figure 2). WBC cell detection: in Experiment A (Figure 1). Bb antibody agglutination: in Experiment A (Figure 1). [file 8835919.f1.zip › Supplementary file/Bb antibody agglutination.pdf]

	Group 1	Group 1	Group 1	Group 1	Group 1	Group 1	Group 1	Group 1
10 day	7.321928	8.321928	7.321928	8.321928	5.321928	9.321928	8.321928	
15 day	5.321928	7.321928	8.321928	7.321928	7.321928	7.321928	9.321928	
21 day	8.321928	7.321928	7.321928	8.321928	7.321928			
35 day	7.321928	7.321928	8.321928	8.321928	6.321928			

	Group 2	Group 2	Group 2	Group 2	Group 2	Group 2	Group 2
10 day	11.32193	10.32193	10.32193	11.32193	11.32193	11.32193	
15 day	12.32193	14.32193	12.32193	12.32193	13.32193	13.32193	12.32193
21 day	13.32193			14.32193	14.32193	13.32193	
35 day	14.32193			14.32193	14.32193	13.32193	

	Group 3	Group 3	Group 3	Group 3	Group 3	Group 3	Group 3
10 day	10.32193	10.32193		10.32193		9.321928	
15 day	12.32193	13.32193	11.32193	13.32193		12.32193	12.32193
21 day	13.32193	13.32193		13.32193	12.32193		
35 day	13.32193	13.32193		13.32193	13.32193		

	Group 4	Group 4	Group 4	Group 4	Group 4	Group 4	Group 4	Group 4
10 day	9.321928	7.321928	5.321928	7.321928	8.321928	7.321928	8.321928	
15 day	6.321928	9.321928	8.321928	8.321928	5.321928	6.321928	9.321928	
21 day	10.32193	9.321928		9.321928	9.321928	9.321928		9.321928
35 day	8.321928	8.321928	7.321928	8.321928	8.321928	9.321928	7.321928	8.321928

	Group 5	Group 5	Group 5	Group 5	Group 5	Group 5	Group 5
10 day	9.321928	8.321928	8.321928	7.321928	7.321928	9.321928	
15 day	9.321928	10.32193	8.321928	9.321928	9.321928	9.321928	11.32193
21 day	9.321928	10.32193	10.32193	7.321928			
35 day	8.321928	9.321928	10.32193	9.321928	10.32193	8.321928	

	Group 6	Group 6	Group 6	Group 6	Group 6	Group 6	Group 6
10 day	8.321928	7.321928	8.321928			6.321928	
15 day	8.321928	9.321928	9.321928		9.321928		7.321928
21 day	8.321928	8.321928	5.321928	6.321928		9.321928	9.321928
35 day	7.321928	5.321928	6.321928		8.321928	8.321928	
