# Supplementary material for: Efficacy of Rg1-Oil Adjuvant on Inducing Immune Responses against Bordetella bronchiseptica in Rabbits
Source: J Immunol Res. 2021 Jan 28;2021:8835919. doi: 10.1155/2021/8835919 (PMC7864750; doi:10.1155/2021/8835919)
Supplement: Supplementary Materials — Concise supplementary material description: W-SCC: in Experiment B (Figure 2). W-MCC: in Experiment B (Figure 2). W-LCC: in Experiment B (Figure 2). WBC-1: in Experiment B (Figure 2). SCC cell detection: in Experiment A (Figure 1). PLT: in Experiment B (Figure 2). OD450nm: in Experiment A (Figure 1). IL-4 35 days postimmunization: in Experiment B (Figure 4). IL-2 35 days postimmunization: in Experiment B (Figure 4). Body weight: in Experiment A (Figure 3). IL-4 15 days postimmunization: in Experiment B (Figure 4). IL-2 15 days postimmunization: in Experiment B (Figure 4). IgG: in Experiment B (Figure 2). WBC cell detection: in Experiment A (Figure 1). Bb antibody agglutination: in Experiment A (Figure 1). [file 8835919.f1.zip › Supplementary file/Body weight.pdf]

	Group 1	Group 1	Group 1	Group 1	Group 1	Group 1
0 day	2.1	2.4	2.3	2	2.1	2.4
5 day	2	2.4	2.3	2	2.1	2.3
10 day	2.3	2.7	2.5	2.2	2.2	2.3
15 day	2.1	2.3	2.3	2.2	2.4	2.4
35 day	2.3	2.8	2.6	2.3	2.5	2.5

	Group 2	Group 2	Group 2	Group 2	Group 2	Group 2
0 day	2.4	2.1	2.2	2	2	
5 day	2.2	2	2.2	2	2	
10 day	2.2	2.1	2.2	2.1	2.1	
15 day	2.2	2.1	2.2	2.2	2.1	
35 day	2.4	2.2	2.4	2.3	2.4	

	Group 3	Group 3	Group 3	Group 3	Group 3	Group 3
0 day	2.2	2.1	2.2	2.1	2.2	2.1
5 day	1.9	2	2	2	2.1	2.1
10 day	2	2.1	2.3	2.2	2.2	2.1
15 day	2.1	2.4	2.2	2.1	2.1	2
35 day	2.4	2.6	2.4	2.3	2.4	2.3

	Group 4	Group 4	Group 4	Group 4	Group 4	Group 4
0 day	2.1	2.1	1.9	2.3	2.1	2.4
5 day	2	1.9	2	2.3	2.1	2.1
10 day	2.3	1.9	2.1	2.4	2.4	2.3
15 day	2.4	2.1	2	2	2.4	2.3
35 day	2.6	2.4	2.5	2.3	2.4	2.6

	Group 5	Group 5	Group 5	Group 5	Group 5	Group 5
0 day	2.2	2.3	2.1	2.2	2.1	2.1
5 day	2	2.1	2.2	2.1	1.9	1.9
10 day	2.2	2.1	2.3	2	2.1	2
15 day	2.1	2	2.4	2.1	2.2	2.4
35 day	2.3	2.5	2.7	2.4	2.4	2.4

	Group 6	Group 6	Group 6	Group 6	Group 6	Group 6
0 day	2	2	2	2.3	2.3	2.5
5 day	2	1.8	1.7	2	2.2	2.3
10 day	2.1	1.8	1.6	2.1	2.3	1.7
15 day	2	1.7	1.5	2.1	2.1	1.8
35 day	2.4	1.9	2	2.4	2.3	2.1
