# Supplementary material for: Efficacy of Rg1-Oil Adjuvant on Inducing Immune Responses against Bordetella bronchiseptica in Rabbits
Source: J Immunol Res. 2021 Jan 28;2021:8835919. doi: 10.1155/2021/8835919 (PMC7864750; doi:10.1155/2021/8835919)
Supplement: Supplementary Materials — Concise supplementary material description: W-SCC: in Experiment B (Figure 2). W-MCC: in Experiment B (Figure 2). W-LCC: in Experiment B (Figure 2). WBC-1: in Experiment B (Figure 2). SCC cell detection: in Experiment A (Figure 1). PLT: in Experiment B (Figure 2). OD450nm: in Experiment A (Figure 1). IL-4 35 days postimmunization: in Experiment B (Figure 4). IL-2 35 days postimmunization: in Experiment B (Figure 4). Body weight: in Experiment A (Figure 3). IL-4 15 days postimmunization: in Experiment B (Figure 4). IL-2 15 days postimmunization: in Experiment B (Figure 4). IgG: in Experiment B (Figure 2). WBC cell detection: in Experiment A (Figure 1). Bb antibody agglutination: in Experiment A (Figure 1). [file 8835919.f1.zip › Supplementary file/IgG.pdf]

	15 days p	15 days p	15 days p	30 days p	30 days p	30 days post immunization
Group1	0.45	0.403	0.499	1.015	0.866	0.695
Group2	0.347	0.369	0.387	0.729	0.814	0.727
Group3	0.349	0.343	0.317	0.637	0.811	0.814
Group4	0.588	0.358	0.47	0.685	0.75	0.734
Group5	0.263	0.214	0.203	0.349	0.332	0.459
Group6	0.126	0.143	0.196	0.228	0.22	0.207
