# Supplementary material for: Efficacy of Rg1-Oil Adjuvant on Inducing Immune Responses against Bordetella bronchiseptica in Rabbits
Source: J Immunol Res. 2021 Jan 28;2021:8835919. doi: 10.1155/2021/8835919 (PMC7864750; doi:10.1155/2021/8835919)
Supplement: Supplementary Materials — Concise supplementary material description: W-SCC: in Experiment B (Figure 2). W-MCC: in Experiment B (Figure 2). W-LCC: in Experiment B (Figure 2). WBC-1: in Experiment B (Figure 2). SCC cell detection: in Experiment A (Figure 1). PLT: in Experiment B (Figure 2). OD450nm: in Experiment A (Figure 1). IL-4 35 days postimmunization: in Experiment B (Figure 4). IL-2 35 days postimmunization: in Experiment B (Figure 4). Body weight: in Experiment A (Figure 3). IL-4 15 days postimmunization: in Experiment B (Figure 4). IL-2 15 days postimmunization: in Experiment B (Figure 4). IgG: in Experiment B (Figure 2). WBC cell detection: in Experiment A (Figure 1). Bb antibody agglutination: in Experiment A (Figure 1). [file 8835919.f1.zip › Supplementary file/OD450nm.pdf]

	Group 1	Group 1	Group 1
0 day	0.150167	0.056148	5
5 day	0.564775	0.098538	5
10 day	0.948633	0.151446	5
15 day	0.8265	0.160782	5
21 day	0.9184	0.0352	5

	Group 2	Group 2	Group 2
0 day	0.2311	0.095787	5
5 day	0.924367	0.084939	5
10 day	2.031333	0.104932	5
15 day	2.281925	0.205963	5
21 day	2.60622	0.134617	5

	Group 3	Group 3	Group 3
0 day	0.176433	0.059127	5
5 day	0.2396	0.083924	5
10 day	1.252433	0.10283	5
15 day	1.846825	0.082828	5
21 day	2.209925	0.220962	5

	Group 4	Group 4	Group 4
0 day	0.129667	0.029238	5
5 day	0.297925	0.09606	5
10 day	0.5751	0.142851	5
15 day	1.153767	0.397665	5
21 day	2.00525	0.506904	5

	Group 5	Group 5	Group 5
0 day	0.1492	0.056055	5
5 day	0.289225	0.091186	5
10 day	0.595133	0.117874	5
15 day	0.66775	0.059207	5
21 day	0.656725	0.12359	5

	Group 6	Group 6	Group 6
0 day	0.187467	0.061647	5
5 day	0.410825	0.195541	5
10 day	0.6208	0.101606	5
15 day	0.707767	0.22233	5
21 day	0.807475	0.324222	5
