# Supplementary material for: Efficacy of Rg1-Oil Adjuvant on Inducing Immune Responses against Bordetella bronchiseptica in Rabbits
Source: J Immunol Res. 2021 Jan 28;2021:8835919. doi: 10.1155/2021/8835919 (PMC7864750; doi:10.1155/2021/8835919)
Supplement: Supplementary Materials — Concise supplementary material description: W-SCC: in Experiment B (Figure 2). W-MCC: in Experiment B (Figure 2). W-LCC: in Experiment B (Figure 2). WBC-1: in Experiment B (Figure 2). SCC cell detection: in Experiment A (Figure 1). PLT: in Experiment B (Figure 2). OD450nm: in Experiment A (Figure 1). IL-4 35 days postimmunization: in Experiment B (Figure 4). IL-2 35 days postimmunization: in Experiment B (Figure 4). Body weight: in Experiment A (Figure 3). IL-4 15 days postimmunization: in Experiment B (Figure 4). IL-2 15 days postimmunization: in Experiment B (Figure 4). IgG: in Experiment B (Figure 2). WBC cell detection: in Experiment A (Figure 1). Bb antibody agglutination: in Experiment A (Figure 1). [file 8835919.f1.zip › Supplementary file/PLT.pdf]

	PLT/(×1(	PLT/(×1(	PLT/(×103/μL)
Group1	613	612	531
Group2	577	576	595
Group3	615	635	633
Group4	561	574	523
Group5	177	280	206
Group6	303	271	286
