# Supplementary material for: Efficacy of Rg1-Oil Adjuvant on Inducing Immune Responses against Bordetella bronchiseptica in Rabbits
Source: J Immunol Res. 2021 Jan 28;2021:8835919. doi: 10.1155/2021/8835919 (PMC7864750; doi:10.1155/2021/8835919)
Supplement: Supplementary Materials — Concise supplementary material description: W-SCC: in Experiment B (Figure 2). W-MCC: in Experiment B (Figure 2). W-LCC: in Experiment B (Figure 2). WBC-1: in Experiment B (Figure 2). SCC cell detection: in Experiment A (Figure 1). PLT: in Experiment B (Figure 2). OD450nm: in Experiment A (Figure 1). IL-4 35 days postimmunization: in Experiment B (Figure 4). IL-2 35 days postimmunization: in Experiment B (Figure 4). Body weight: in Experiment A (Figure 3). IL-4 15 days postimmunization: in Experiment B (Figure 4). IL-2 15 days postimmunization: in Experiment B (Figure 4). IgG: in Experiment B (Figure 2). WBC cell detection: in Experiment A (Figure 1). Bb antibody agglutination: in Experiment A (Figure 1). [file 8835919.f1.zip › Supplementary file/SCC cell detection.pdf]

	Group 1	Group 1	Group 1	Group 1	Group 1
10 day	4.7	7.1	4	7	9.5
15 day	6		6.9	6	6.4
21 day	7.9	6.4	6.3	5.4	
35 day	8.5	7.3	9	7.8	9.1

	Group 2	Group 2	Group 2	Group 2	Group 2
10 day	8.8	4.1	4.1	5.8	7.9
15 day	7	6.7	6.9	8.9	7.6
21 day	9.9	8.7	8.3		8
35 day	7.7	11.1	8.8	7.6	10.6

	Group 3	Group 3	Group 3	Group 3	Group 3
10 day	5.1	3.5	4.6	9.2	5.5
15 day	8.9	7.3	6.3		6.2
21 day	7.6	7.3	4.5	7.4	3.8
35 day	7.7	5.5	7.5		5.9

	Group 4	Group 4	Group 4	Group 4	Group 4
10 day	3.3	5			4.6
15 day	4.3	6.5	6.5		
21 day	6	6.6		4.6	5.2
35 day	5.7	6.4	3.7		4.8

	Group 5	Group 5	Group 5	Group 5	Group 5	Group 5
10 day	3.9	5.9	4.1	3.5		
15 day	3.8	6.1	6.4	7.6	4.9	
21 day	4.3	4.5	4.9	5.4	4.5	
35 day	5.3	9.7	5	6.3	4.4	8.7

	Group 6	Group 6	Group 6	Group 6	Group 6	Group 6
10 day	2.5	2	5.6	4.3		
15 day	3.4	4.3	5.7	4.5	4.3	
21 day	6.1	5.4	4.3	4.2	4.9	
35 day	5.9	6.6		5.1	6.1	7.4
