# Supplementary material for: Efficacy of Rg1-Oil Adjuvant on Inducing Immune Responses against Bordetella bronchiseptica in Rabbits
Source: J Immunol Res. 2021 Jan 28;2021:8835919. doi: 10.1155/2021/8835919 (PMC7864750; doi:10.1155/2021/8835919)
Supplement: Supplementary Materials — Concise supplementary material description: W-SCC: in Experiment B (Figure 2). W-MCC: in Experiment B (Figure 2). W-LCC: in Experiment B (Figure 2). WBC-1: in Experiment B (Figure 2). SCC cell detection: in Experiment A (Figure 1). PLT: in Experiment B (Figure 2). OD450nm: in Experiment A (Figure 1). IL-4 35 days postimmunization: in Experiment B (Figure 4). IL-2 35 days postimmunization: in Experiment B (Figure 4). Body weight: in Experiment A (Figure 3). IL-4 15 days postimmunization: in Experiment B (Figure 4). IL-2 15 days postimmunization: in Experiment B (Figure 4). IgG: in Experiment B (Figure 2). WBC cell detection: in Experiment A (Figure 1). Bb antibody agglutination: in Experiment A (Figure 1). [file 8835919.f1.zip › Supplementary file/W-LCC.pdf]

	W-LCC/(×	W-LCC/(×	W-LCC/(×103/μ L)
Group1	1.7	2.1	1.7
Group2	2	2.6	1.6
Group3	3.1	2.1	1.9
Group4	1.5	2.9	1.9
Group5	0.8	0.5	0.5
Group6	0.3	0.3	0.4
