# Supplementary material for: Efficacy of Rg1-Oil Adjuvant on Inducing Immune Responses against Bordetella bronchiseptica in Rabbits
Source: J Immunol Res. 2021 Jan 28;2021:8835919. doi: 10.1155/2021/8835919 (PMC7864750; doi:10.1155/2021/8835919)
Supplement: Supplementary Materials — Concise supplementary material description: W-SCC: in Experiment B (Figure 2). W-MCC: in Experiment B (Figure 2). W-LCC: in Experiment B (Figure 2). WBC-1: in Experiment B (Figure 2). SCC cell detection: in Experiment A (Figure 1). PLT: in Experiment B (Figure 2). OD450nm: in Experiment A (Figure 1). IL-4 35 days postimmunization: in Experiment B (Figure 4). IL-2 35 days postimmunization: in Experiment B (Figure 4). Body weight: in Experiment A (Figure 3). IL-4 15 days postimmunization: in Experiment B (Figure 4). IL-2 15 days postimmunization: in Experiment B (Figure 4). IgG: in Experiment B (Figure 2). WBC cell detection: in Experiment A (Figure 1). Bb antibody agglutination: in Experiment A (Figure 1). [file 8835919.f1.zip › Supplementary file/_WBC cell detection.pdf]

	Group 1	Group 1	Group 1	Group 1	Group 1
10 day	15.3	13.2	10.2	16.7	
15 day	10.3	10.6	10.7		
21 day	10.8	9.2	12.1	9.7	
35 day	10.3	10.6	11.6	10.6	

	Group 2	Group 2	Group 2	Group 2	Group 2
10 day	12.9	13	12		
15 day	14	11.4	14.2	15	15
21 day	13.5	11	11.3	11.5	11.5
35 day	18.3	14.5	15.3	15.4	15.1

	Group 3	Group 3	Group 3	Group 3
10 day	8.4	11.4	13.4	
15 day	11.3	11.1	13	
21 day	11.8	11.1	12.4	
35 day	12.3	10	12.3	10.1

	Group 4	Group 4	Group 4	Group 4	Group 4
10 day	14.3	11.4	13.2	9.5	
15 day	10.5	11.5	11		
21 day	6.5	6.9	9.3	7	9.1
35 day	6.8	7.9	7.6	6.1	

	Group 5	Group 5	Group 5	Group 5	Group 5
10 day	6.2	8.7	6.6	7.4	
15 day	5.2	7.5	8.1		
21 day	5.7	5.2	6.5	7.1	7.2
35 day	6.8	7	8.1		

	Group 6	Group 6	Group 6	Group 6	Group 6
10 day	3.6	3.1	5.7		
15 day	4.9	5.2	7.8	5.5	5.1
21 day	7.9	5.5	5.3	6.1	
35 day	7.6	6.7	7		
